# The Relationship between White Matter Architecture and Language Lateralization in the Healthy Brain

**DOI:** 10.1523/JNEUROSCI.0166-24.2024

**Published:** 2024-10-07

**Authors:** Ieva Andrulyte, Christophe De Bezenac, Francesca Branzi, Stephanie J. Forkel, Peter N. Taylor, Simon S. Keller

**Affiliations:** ^1^Departments of Pharmacology and Therapeutics, Institute of Systems, Molecular and Integrative Biology, University of Liverpool, Liverpool L3 9TA, United Kingdom; ^2^Psychological Sciences, Institute of Population Health, University of Liverpool, Liverpool L3 9TA, United Kingdom; ^3^Donders Institute for Brain Cognition Behaviour, Radboud University, Nijmegen, The Netherlands; ^4^Brain Connectivity and Behaviour Laboratory, Sorbonne Universities, Paris, France; ^5^Department of Neuroimaging, Centre for Neuroimaging Sciences, Institute of Psychiatry, Psychology and Neuroscience, King’s College London, London, United Kingdom; ^6^CNNP Lab (www.cnnp-lab.com), Interdisciplinary Computing and Complex BioSystems Group, School of Computing Science, Newcastle University, Newcastle, United Kingdom; ^7^Institute of Neurology, Queen Square, University College London (UCL), London, United Kingdom

**Keywords:** connectometry, corpus callosum, forceps minor, hemispheric asymmetries, language lateralization, MRI, tractography, white matter asymmetries

## Abstract

Interhemispheric anatomical differences have long been thought to be related to language lateralization. Previous studies have explored whether asymmetries in the diffusion characteristics of white matter language tracts are consistent with language lateralization. These studies, typically with smaller cohorts, yielded mixed results. This study investigated whether connectomic analysis of quantitative anisotropy (QA) and shape features of white matter tracts across the whole brain are associated with language lateralization. We analyzed 1,040 healthy individuals (562 females) from the Human Connectome Project database. Hemispheric language dominance for each participant was quantified using a laterality quotient (LQ) derived from fMRI activation in regions of interest (ROIs) associated with a language comprehension task compared against a math task. A linear regression model was used to examine the relationship between structural asymmetry and functional lateralization. Connectometry revealed a significant negative correlation between LQs and QA of corpus callosum tracts, indicating that higher QA in these regions is associated with bilateral and right hemisphere language representation in frontal and temporal regions. Left language laterality in the temporal lobe was significantly associated with longer right inferior fronto-occipital fasciculus (IFOF) and forceps minor tracts. These results suggest that diffusion measures of microstructural architecture as well as geometrical features of reconstructed white matter tracts play a role in language lateralization. People with increased dependence on the right or both frontal hemispheres for language processing may have more developed commissural fibers, which may support more efficient interhemispheric communication.

## Significance Statement

The left cerebral hemisphere is dominant for language functions in most people. In some healthy people, language functions are lateralized to the right hemisphere or distributed across both hemispheres. The anatomy underlying patterns of hemispheric language dominance are not well established. Emerging evidence suggests that white matter connectivity and architecture are important features of cortical functional organization. In this work, we report that people who have language functions distributed across both hemispheres have greater interhemispheric connectivity compared with lateralized people. Our findings provide further insights into the anatomical basis of language function and may have wider clinical implications.

## Introduction

It has long been hypothesized that gray matter asymmetries of regions that support language function may be associated with the functional lateralization of language in the human brain (Güntürkün et al., 2020). Associations between cortical asymmetries and hemispheric language dominance (HLD) have been noted in some studies of Wada-tested patients with epilepsy (Foundas et al., 1996; Dorsaint-Pierre et al., 2006; Keller et al., 2018) and healthy controls who underwent structural and functional MRI (Josse et al., 2009; Keller et al., 2011). However, other studies have reported no association between HLD and structural hemispheric asymmetry in classical language cortical areas (Chiarello et al., 2013; Greve et al., 2013). Attention has recently shifted toward the importance of white matter as the basis of lateralized cortical function. Some studies have reported that leftward language lateralization is associated with a greater volume of the arcuate fasciculus (Propper et al., 2010) and the number of tracts in the corpus callosum (Timocin et al., 2020). More recently, HLD has been investigated using microstructural DTI properties, such as fractional anisotropy (FA), in patient cohorts (Tantillo et al., 2016; Barba et al., 2020). Some studies have reported relationships between language lateralization and diffusion characteristics or the size of the corpus callosum (Tantillo et al., 2016), while others did not (Barba et al., 2020). There are limited functional MRI (fMRI)-DTI studies on language lateralization in healthy individuals. Tractography studies have reported associations between fMRI-determined left HLD and FA of the left arcuate fasciculus (Powell et al., 2006; Perlaki et al., 2013; James et al., 2015; Silva and Citterio, 2017) and corpus callosum (Häberling et al., 2011). However, other studies have not reported relationships between the side or extent of HLD and conventional diffusion-based tract characteristics (Vernooij et al., 2007; Karpychev et al., 2022), as well as more sophisticated diffusion MRI measures, such as fiber density cross section (Verhelst et al., 2021). Inconsistencies between studies may be due to methodological differences, including differences in tractography approaches, study designs, patient characteristics, and sample sizes.

In the present study, we adopted two complementary approaches that potentially overcome some of the methodological shortcomings of previous tractography studies. First, we employed a connectometry approach based on a local analysis of diffusion properties, which uses permutation testing to identify group differences along white matter tracts. This whole-brain approach employs correlational tractography to identify specific subcomponents of white matter tracts that exhibit anisotropy correlated with a predefined variable of interest with superior sensitivity and specificity compared with traditional voxel-based analyses (Yeh et al., 2016). Connectometry has recently been used to uncover structural disparities between bilingual and nonbilingual individuals (Rahmani et al., 2017) and to identify structural pathways linked to enhanced language capabilities in individuals with aphasia (Hula et al., 2020; Dresang et al., 2021) and preterm-born children (Barnes-Davis et al., 2020, 2022). Second, we employed shape analysis to investigate the geometrical characteristics of white matter tract bundles that comprise the integral components of language networks. This approach captures fundamental shape characteristics, such as volume and surface area, and encompasses advanced morphological properties including white matter bundle curl, elongation, length, span, and diameter (Yeh, 2020). Previous studies have already demonstrated, through the utilization of virtual dissections (Catani et al., 2007) and shape analysis employing tractography algorithms (Yeh, 2020), that the leftward morphometric asymmetries of language-associated white matter tracts exist in people without known hemispheric language dominance (HLD). Whether white matter interhemispheric asymmetries change in people with atypical HLD remains unclear.

The first objective of the present study was to conduct diffusion connectometry analysis in a large cohort of healthy individuals who underwent language fMRI to determine whether microstructural properties of white matter tracts are related to HLD. The second objective was to explore whether interhemispheric shape asymmetries of white matter tracts are related to language lateralization in the same individuals.

## Materials and Methods

### Study data and participants

All data were acquired from the Human Connectome Project (HCP; http://www.humanconnectome.org/) open-access data initiative offering high-quality anatomical and functional MRI of the human brain. We used the HCP Young Adults (HCP-YA 1200 Subjects) data release as it contains a large sample of healthy adults for whom both language task fMRI and diffusion MRI sequences were acquired. The dataset comprised 1,200 healthy adults, aged 22–35 years. Each participant underwent an identical imaging protocol acquired on the same MRI scanner. Individuals with neuropsychiatric or neurologic disorders, diabetes, high blood pressure, premature birth, and severe symptoms associated with substance use were excluded from data collection (Van Essen et al., 2013). The present study focused on language fMRI and diffusion MRI data only. Individuals were only selected for inclusion if they had fMRI data available for the language story task (see below) and had corresponding 3T diffusion MRI data. This resulted in a sample size of 1,040 participants (562 females), with a mean age of 28.74 (SD = 3.69) years. According to the Edinburgh Handedness Inventory (Oldfield, 1971), 962 (92%) participants preferred their right hand, scoring at least 10 on a scale of −100 (left) to 100 (right). Eighty-five participants preferred left, scoring below −10, and two were ambidextrous, scoring zero.

### Data acquisition

HCP data were acquired on a Siemens 3T Skyra system, with a 32-channel (SC72) head coil. Task fMRI data were collected using gradient-echo echo-planar imaging (EPI) with an isotropic resolution of 2.0 mm (TR, 720 ms; TE, 33.1 ms; matrix, 104 × 90, 72 slices; flip angle, 52°; BW, 2,290 Hz/Px; FOV, 208 × 180 mm, 72 slices; multiband accelerator factor, 8; Marcus et al., 2013). The HCP dMRI data were acquired using three shells (*b* = 1,000, 2,000, and 3,000 s/mm^2^) with 90 diffusion gradient directions and five *b*_0_ volumes with RL phase encoding direction (TE, 89.5 ms; TR, 5,520 ms; flip angles, 78/160°; isotropic voxel size, 1.25 mm^3^, multiband factor, 3; Sotiropoulos et al., 2013). A list of technical abbreviations is provided in Table 1.

### Language paradigm

The language comprehension task used in the Human Connectome Project was designed by Binder et al. (2011). The task consists of two 3.8 min runs. Each run has four blocks of story tasks alternating with four blocks of math tasks. The story and math tasks are matched in terms of length, word and phoneme rate, speaking style, and prosodic features. The story blocks present subjects with 5–9 auditory sentences, followed by questions about the content of the story. The math task requires participants to perform arithmetic operations followed by equals and two choices. Since arithmetic tasks do not engage temporal lobe activity (Baldo and Dronkers, 2007), we decided to use a STORY–MATH contrast, as it effectively isolates regions responsible for language comprehension without “masking” temporal lobe activity. Additionally, the temporal lobe is involved in high-level processes of normal consciousness (Spitsyna et al., 2006); thus, we avoided passive tasks as a baseline to reduce the risk of masking activities in this region (which is essential for language comprehension). This contrast allowed us to cancel out the regions that are jointly activated in both tasks (such as low-level auditory and phonological input), isolating the regions involved in narrative processing including semantic and nonspeech-related aspects of language, theory of mind, and inference processing.

### fMRI preprocessing and analysis

The preprocessed task fMRI data were retrieved from the HCP database (https://db.humanconnectome.org). The HCP preprocessing included *fMRIVolume* and *fMRISurface* pipelines, which were primarily built using tools from FSL (Jenkinson et al., 2012; http://www.fmrib.ox.ac.uk/fsl), FreeSurfer (Fischl, 2012), and the HCP Workbench (Marcus et al., 2013). Details of the preprocessing steps have been described previously (Glasser et al., 2013). The goal of the first *fMRIVolume* pipeline was to generate a 4D whole-brain time series. This was accomplished by (1) removing spatial distortions by gradient nonlinearity distortion correction, (2) realigning volumes using rigid-body motion correction using a single-band reference image as the target, and (3) estimating (using FSL toolbox “topup”) and correcting field map-based EPI distortions. The resulting EPI data were (4) registered to a T1-weighted scan and then (5) nonlinearly (FNIRT) to Montreal Neurological Institute (MNI) space, and (6) blood oxygenation level−dependent (BOLD) signal intensity was normalized by the average. This process resulted in individual subjects being mapped with a notable degree of left–right symmetry (Elam et al., 2021), which aligns with laterality research recommendations (Vingerhoets et al., 2023).

The goal of *fMRISurface* pipeline was to transform the resulting 4D time series into Connectivity Informatics Technology Initiative (CIFTI) grayordinate space, encompassing cortical, subcortical, and cerebellar gray matter collectively (Pham et al., 2022). This was accomplished by mapping fMRI data within cortical gray matter ribbon onto the native cortical surface, registering it to CIFTI grayordinate space (surface representation with 32,492 vertices on each hemisphere), and mapping the set of subcortical gray matter voxels from each subcortical parcel in each individual to a standard set of voxels in each atlas parcel, resulting in 2 mm average surface vertex and subcortical volume voxel spacing. Finally, grayordinate space data were smoothed using the Gaussian kernel.

We used a fully processed task-based STORY–MATH fMRI activation contrast of parameter estimates (COPE) map, which was generated by FSL FEAT and is readily available on https://db.humanconnectome.org as part of the “S1200 Subjects” dataset. Considering the spatial heterogeneity of the individual brain scans, the MSM-ALL (Multimodal Surface Matching) registered dataset was used, which uses information on areal features derived from the resting state network, myelin maps, and alignment of folding. The motivation for using MSM-ALL over MSM-SULC (cortical folding-based registration) came from previous studies that demonstrated a weaker correlation between sulcal depth and local curvature with regions responsible for higher cognitive functions, including Broca's area (Van Essen, 2005; Fischl et al., 2008), compared with the MSM-ALL registration, which showed improved cross-subject alignment of independent task fMRI datasets (Robinson et al., 2018).

### Language comprehension laterality quotient

**Figure 1.**
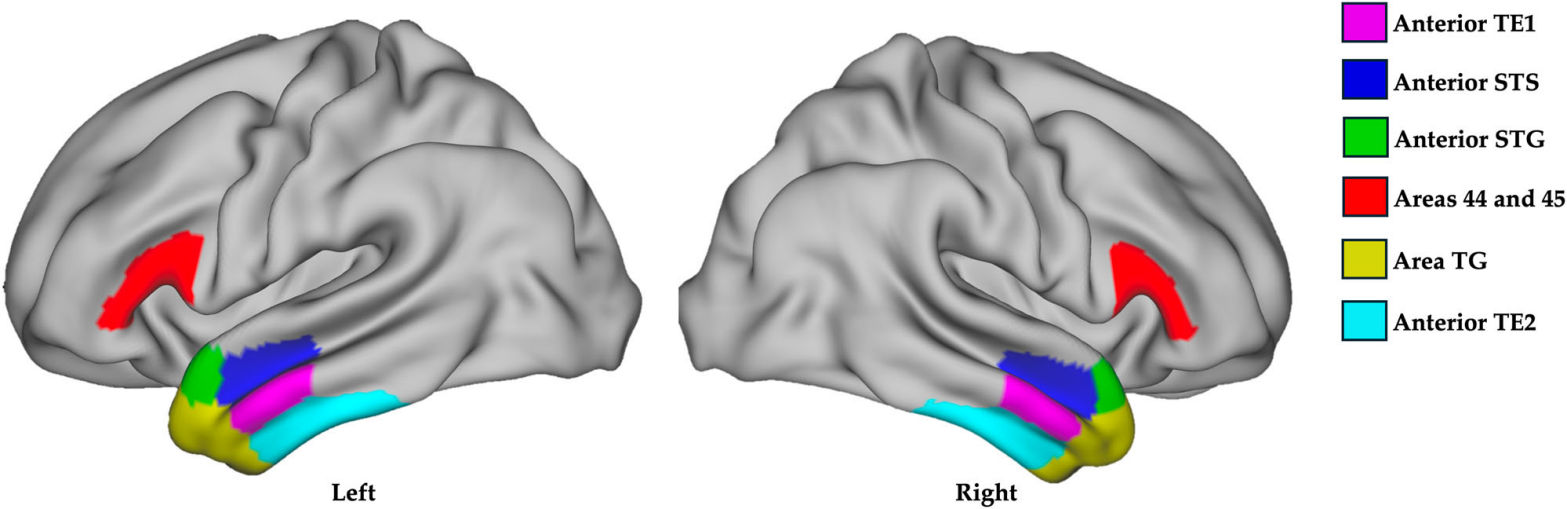
Regions of interest (ROIs) were selected to calculate LQ based on the Jaccard–Tanimoto index (more details provided in text; Seghier, 2019). The LQ value is expressed as a percentage, ranging from −1 to 1. Values greater than 1/3 indicate left lateralization, values less than −1/3 indicate right lateralization, and values between −1/3 and 1/3 indicate bilateral orientation (Seghier, 2019). TE1, temporal area 1, found in middle temporal gyrus; STS, superior temporal sulcus; STG, superior temporal gyrus; TG, temporal gyrus; TE2, temporal area, including ventral and dorsal parts of inferior temporal gyrus.

Grayordinates localized regions of interest (ROIs) on the “inflated” brain surface (Glasser et al., 2016; Van Essen and Glasser, 2016). A laterality quotient (LQ) was calculated to assess HLD for each participant's task fMRI activation using the CIFTI toolbox in MATLAB in ROIs associated with language comprehension. The analyses were conducted separately for frontal and temporal regions, given the well-documented phenomenon of crossed language dominance, where a participant may exhibit dominance in one hemisphere for frontal regions and the opposite hemisphere for temporal regions (Seghier, 2008). For the frontal ROIs, Brodmann areas 44 and 45 were selected due to their established high reliability in determining language dominance during semantic tasks (Sabbah et al., 2003; Seghier et al., 2008). In our temporal lobe laterality analyses, ROIs were chosen within the anterior temporal lobe (TGd, TGv, TE1a, TE2a, STGa, STSva, STSda) because these areas have been shown to be heavily involved in language comprehension (Binder et al., 2011; Fig. 1).

To work with CIFTI files, we generated *dscalar* files for each ROI using *wb_command*, imported them into MATLAB, and extracted *z*-values from ROIs using the CIFTI toolbox. The *z*-values were thresholded for each participant by including only grayordinates with values greater than the median in each ROI (Dietz et al., 2016). To account for the unequal number of grayordinates between the left and right hemispheres (approximately 100 more grayordinates on the left than the right), we corrected these regional differences to ensure that comparisons between hemispheres were not skewed by differences in their sizes. This adjustment involved dividing the total sum of thresholded *z*-values by the number of grayordinates in each hemisphere for both frontal and temporal ROIs separately. The laterality quotient (LQ) was then computed for each participant's normalized *z*-values using the equations below:

$$LQ=\;\frac{\left(L-R\right)}{\max(L,R)}$$

where *L* and *R* represent the normalized *z*-values in the left and right ROIs, respectively. We chose to employ an innovative LQ formula based on the Jaccard–Tanimoto index to provide a more sophisticated approach in evaluating and classifying language lateralization (Seghier, 2019). This revised formula defines LQ as a metric of distance that adheres to a consistent distribution pattern, thus enhancing its sensitivity toward hemisphere activity differences, accentuating the distinctions between the two hemispheres. The values above +1/3 indicate left language dominance (LLD), values below −1/3 indicate right language dominance (RLD), and values between −1/3 and +1/3 indicate bilateral language representation (BLR), ensuring an equal cumulative probability in each dominance category.

### Diffusion processing

**Figure 2.**
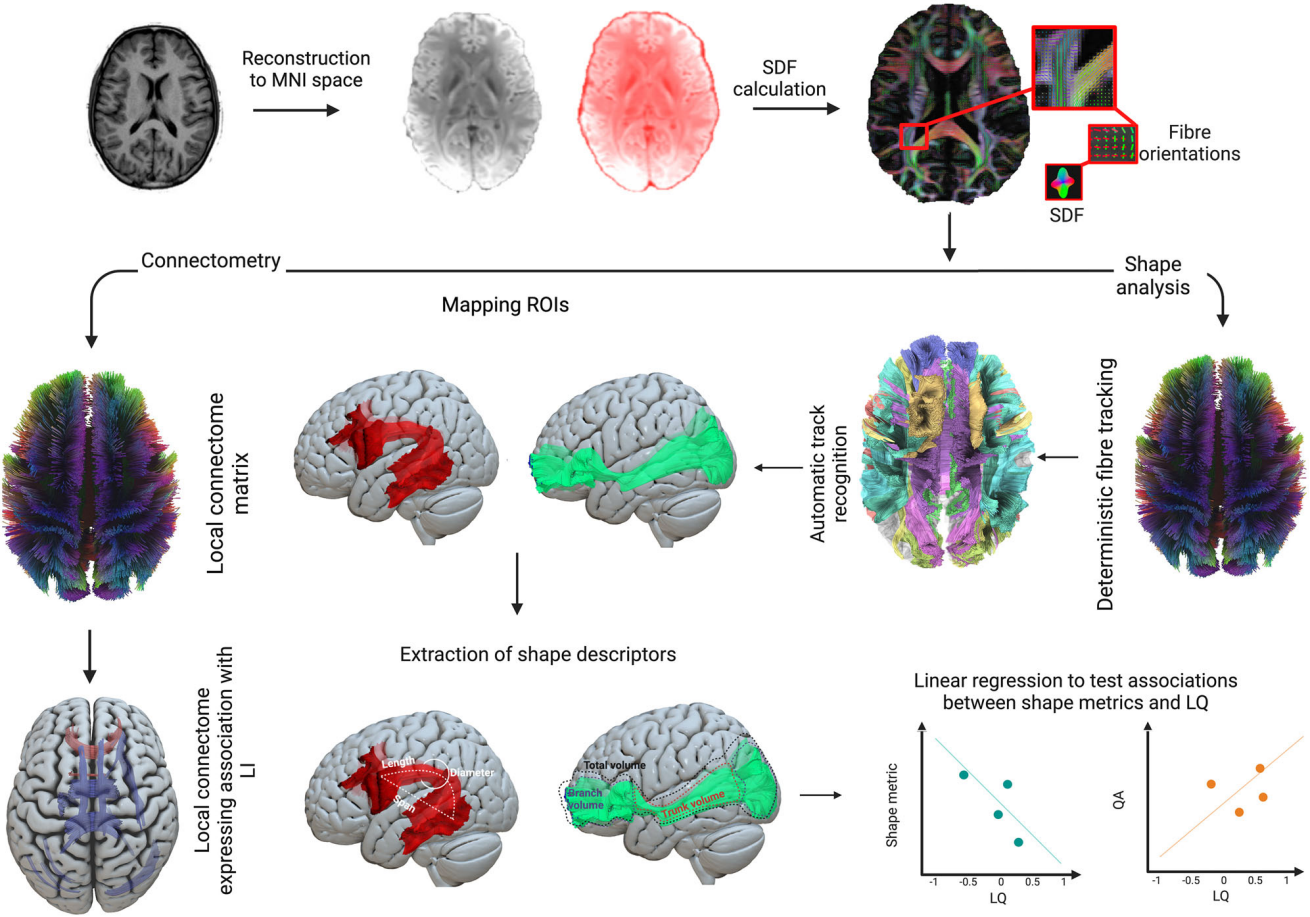
Flowchart of the methods pipeline. The preprocessed diffusion MRI data were reconstructed in an MNI space. The outputs of the reconstruction and SDFs were calculated to obtain the fiber orientations using DSI Studio. Then, two different approaches were used to examine the white matter tracts associated with language laterality. The connectometry approach involved obtaining a local connectome matrix and finding out its association with LQ. Shape analysis involved the recognition of the WM tracts using the HCP atlas and mapping eleven WM fiber bundles important for language function. The measures of key shape features, such as curl and volume, were extracted and linear regression analyses were used to look at the associations between shape metrics and LQ.

Diffusion data were downloaded from the HCP S1200 Young Adult Data Release and preprocessed using the HCP Diffusion preprocessing pipeline using the FMRIB diffusion toolbox in FSL. Briefly, the pipeline included b_0_ image intensity normalization, removing EPI susceptibility-induced field distortions with FSL's “topup” algorithm (Andersson and Sotiropoulos, 2016), correcting for eddy current distortions, head movements, and gradient nonlinearities (Glasser et al., 2013). Quality control of the preprocessed diffusion MRI data was performed using DSI Studio software (http://dsi-studio.labsolver.org). An automatic quality control routine then checked the b-table to ensure its accuracy (Schilling et al., 2019). The diffusion data were coregistered in MNI space using q-space diffeomorphic reconstruction (Yeh and Tseng, 2011) to obtain the spin distribution function (SDF) with a recommended length ratio of 1.25, as specified in the original study (Yeh et al., 2010; Fig. 2).

### Connectometry analysis

We applied a whole-brain group connectometry analysis using DSI Studio as described in previous applications (Rahmani et al., 2017; Barnes-Davis et al., 2020, 2022; Dresang et al., 2021) to study the relationship between regional white matter quantitative anisotropy (QA) and language lateralization measures derived from LQs (Fig. 2). The connectometry approach derives the QA measure from the SDF in each fiber orientation, which defines the number of anisotropic spins along that direction in each streamline (Yeh et al., 2010, 2013). The anisotropy in each section of a white matter tract is then correlated with the study variable (Yeh et al., 2016). Unlike a voxel-based FA metric, which attributes identical anisotropy values to all fiber orientations within a voxel, QA demonstrates a discerning capability by identifying specific axonal orientations in each peak orientation of the SDF (Yeh et al., 2013).

Our connectometry analyses were conducted in two phases: the initial phase focused on examining the lateralization of frontal regions during language comprehension and the subsequent phase investigated temporal regions. Initially, connectometry analyses were performed on all participant groups concurrently, followed by post hoc analyses on three distinct groups separately to aid in interpretation and capture varying effects related to different degrees of laterality. Specifically, the first post hoc analysis included participants with LLD and BLR, the second consisted of participants with RLD and BLR, and the third included individuals with both LLD and RLD. The linear effect of handedness, sex, and age was mitigated using a partial linear correlation. A nonparametric Spearman partial correlation was used to derive the continuous segments correlating with an LQ (Yeh et al., 2016). Each reconstructed white matter tract within a voxel was tracked to extract a QA map for each participant (Yeh et al., 2013). A *T*-score threshold was assigned to the highest level of three to reduce the possibility of false positive results (Ashraf-Ganjouei et al., 2019). The tracks were filtered by topology-informed pruning with 16 iterations to remove implausible spurious connections (Yeh et al., 2019). Given the large sample size in our study, and to prevent false positives, a conservative false discovery rate (FDR) correction for multiple comparisons was employed with a threshold of 0.01 to select tracks showing significant associations between LQ and QA. To estimate the false discovery rate, 5,000 randomized permutations were applied to the group label to obtain the null distribution of the track length. After the correlational results were obtained, additional categorical analyses were performed at the group level (LLD/RLD, LLD/BLR, RLD/BLR). Short tracts (<20 mm) were removed for easier interpretation of our results.

### Shape analysis

**Figure 3.**
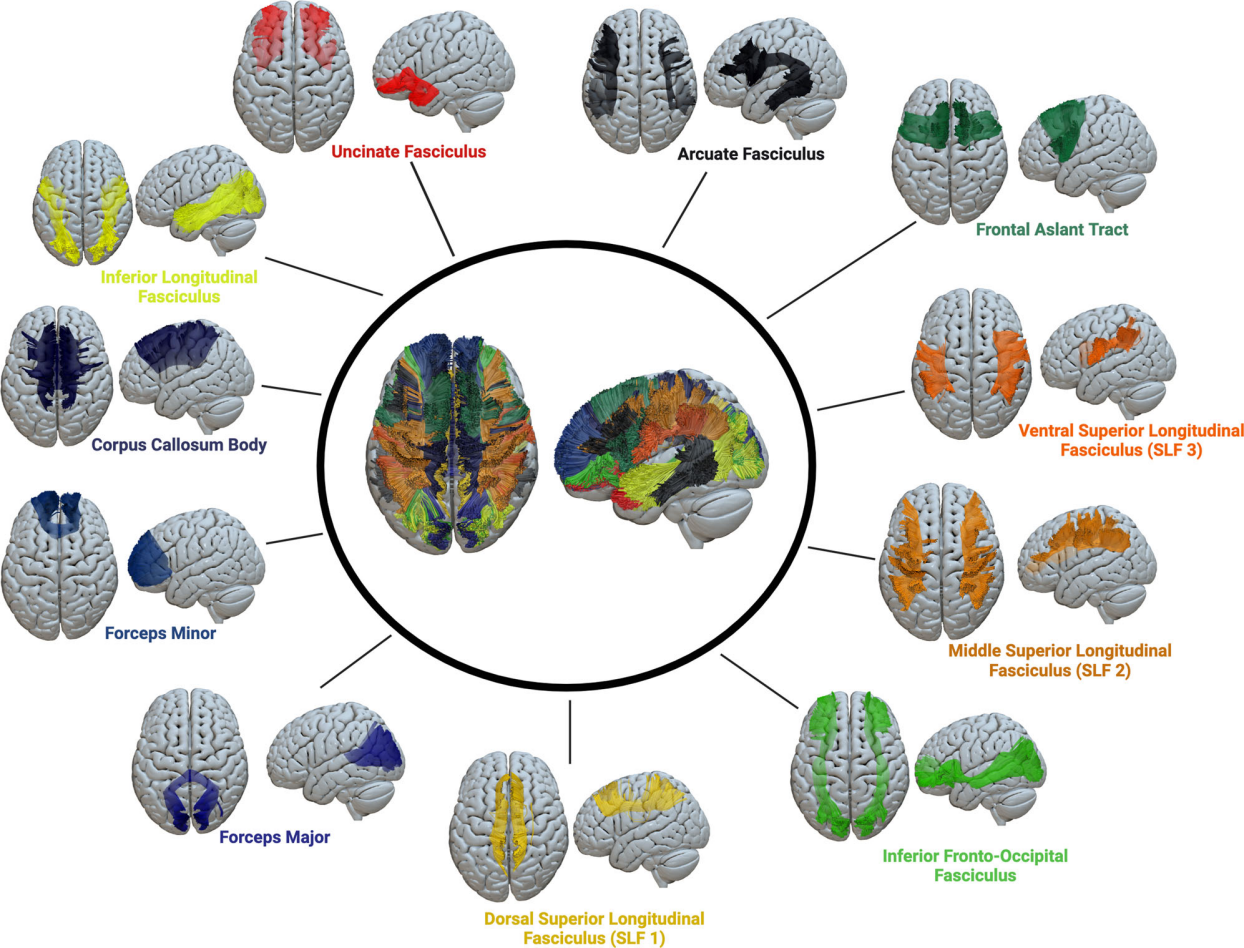
Eleven white matter tracts were reconstructed for shape analysis based on the HCP-842 atlas computed on 1,065 healthy people (Yeh et al., 2018).

**Figure 4.**
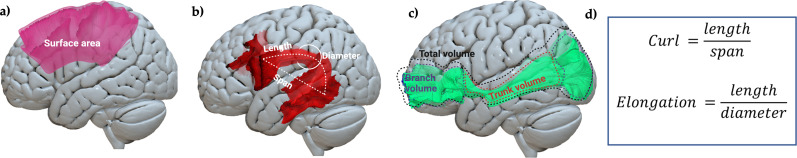
Schematic illustration of the shape analysis of the white matter tracts. ***a***, The area metrics used in the included surface area (mm). ***b***, The length metrics used in the study included mean tract length (mm) as well as span bundle (mm) and diameter (mm) of the bundle. ***c***, The volume metrics used in the study included branch volume (mm^3^; blue dotted line), trunk volume (mm^3^; right dotted line), and total bundle volume (mm^3^; black dotted line). ***d***, The shape metrics used in the study included curl and elongation.

The SDF maps generated from the connectometry analysis were used for tract shape analysis, and automatic fiber tractography was performed using a deterministic fiber tracking algorithm utilizing DSI Studio software (Yeh, 2020). Eleven white matter tract bundles that are part of language comprehension networks (Friederici et al., 2007; Harvey et al., 2013; Rollans and Cummine, 2018; Shin et al., 2019; Ivanova et al., 2021; Forkel et al., 2022; Zhong et al., 2022) were then automatically tracked and recognized based on the HCP-842 tractography atlas (Yeh et al., 2018; Fig. 3). These include the arcuate fasciculus (AF), corpus callosum body, corpus callosum forceps major (splenium), corpus callosum forceps minor (genu), inferior fronto-occipital fasciculus (IFOF), frontal aslant tract (FAT), inferior longitudinal fasciculus (ILF), the three branches of the superior longitudinal fasciculus (dorsal SLF1, middle SLF2, and ventral SLF3), and the uncinate fasciculus. All white matter bundles were independently tracked within the left and right hemispheres, while the corpus callosum bundles were tracked as a whole. The diffusion sampling length ratio was set at 1.25, and the output resolution was resampled to 2 mm isotropic. To remove false connections, topology-informed pruning was applied with 32 iterations (Yeh et al., 2019). We decided to exclude participants for whom we could not reconstruct at least one of their ROI bundles. As a result, 290 participants were excluded, leaving us with a final sample size of 750 participants. Finally, after identifying all white matter tracts of interest, the following shape metrics were extracted: tract length, span, curl, elongation, diameter, volume, and surface area were extracted (Fig. 4).

To evaluate the statistical significance of differences among various laterality groups (LLD, RLD, BLR), we conducted an analysis of variance (ANOVA). This analysis utilized the same laterality groupings based on frontal and temporal ROIs and included covariates consistent with those used in the connectometry analyses. All computations were performed using R (version 4.4.1).

## Results

### fMRI

**Figure 5.**
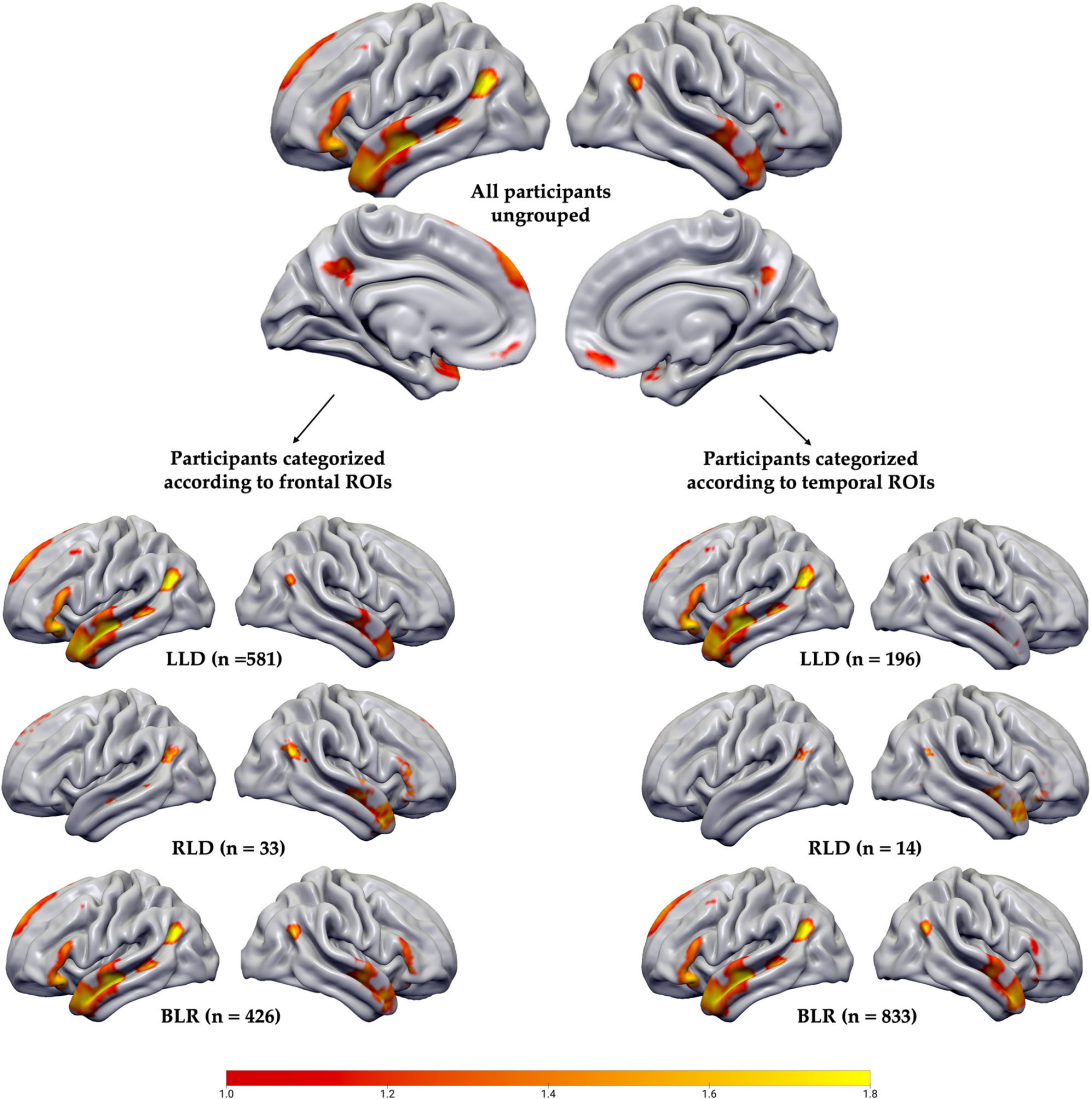
*Z*-score maps of language comprehension task. The color bar indicates *Z* scores; L, left hemisphere; R, right hemisphere.

Activations in frontal ROIs revealed a weak leftward lateralization on the group level (LQ = 0.33 ± 0.31), while BOLD activations in anterior temporal lobe ROIs showed a more bilateral pattern (LQ = 0.17 ± 0.2). Based on the frontal ROIs of the fMRI language comprehension task, 581 participants were classified as left hemisphere dominant (56%; LQ = 0.53 ± 0.16), 426 as bilateral (41%; LQ = 0.13 ± 0.17), and the remaining 33 as right hemisphere dominant (3%; LQ = −0.64 ± 0.17). For temporal ROIs, only 193 participants (19%; LQ = 0.41 ± 0.09) were left hemisphere dominant, while 80% (*n* = 833) were classified as bilateral (LQ = 0.13 ± 0.14). Only 14 participants (1%; LQ = −0.58 ± 0.28) were right lateralized. In both laterality groups, strong neural activity is observed in brain areas associated with semantic processing, i.e., anterior and posterior temporal lobes, as well as in the left inferior frontal gyrus (Jackson, 2021; Fig. 5). In line with previous reports that have used similar story/narrative materials, also the ventral angular gyrus bordering the temporoparietal junction was engaged during language comprehension (Lerner et al., 2011; Humphreys and Lambon Ralph, 2015; Branzi et al., 2020, 2021).

### Connectometry

**Figure 6.**
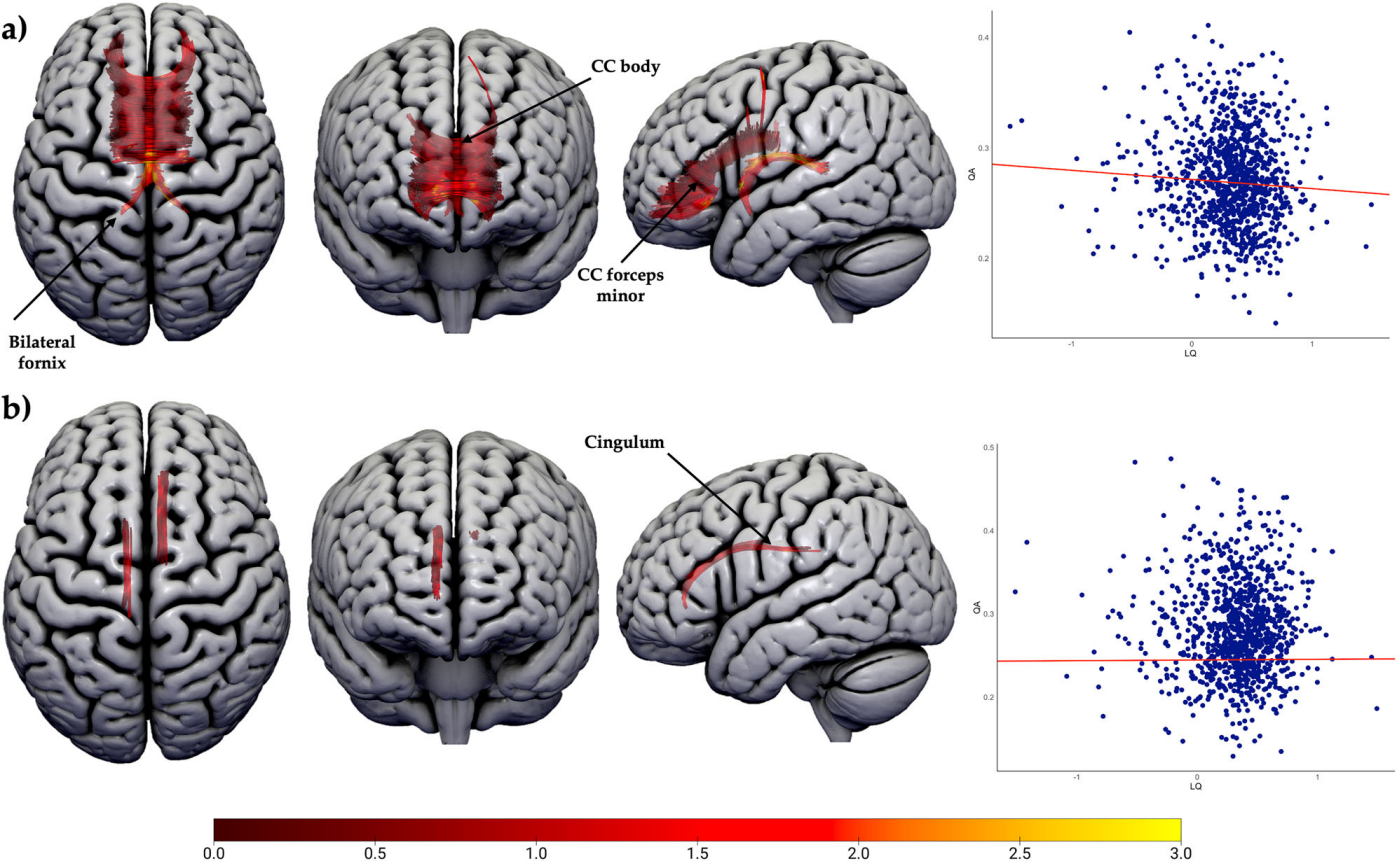
Connectometry results for the language lateralization in the frontal lobe (*n* = 1,040). ***a***, Tract sections negatively correlated with LQ (*p* < 0.01, FDR corrected). ***b***, Tract sections positively correlated with LQ (*p* < 0.01, FDR corrected). Abbreviations: CC, corpus callosum. The color bar represents the *t*-statistic. See Extended Data Figure 6-1 for the group-level post hoc analyses.

Two connectometry analyses were conducted. Firstly, in the investigation of language lateralization in frontal regions, a total of 2,028 white matter tracts exhibiting a significant negative correlation with LQ (*n* = 1,040; *p* < 0.01, FDR corrected) were identified (Fig. 6). Notably, the majority of these tracts were commissural, constituting 82% of the identified tracts, including the forceps minor (63%) and corpus callosum body (19%). A smaller portion of these tracts were positioned in bilateral fornix (18%). Conversely, 218 tracts were linked to higher frontal LQ values, all of which were situated in bilateral cingulum (*n* = 1,040; *p* < 0.01, FDR corrected). Subsequently, categorical post hoc analyses were conducted to further illustrate the laterality groups influencing significant differences, with a specific focus on the disparities between LLD and BLR, RLD and BLR, and RLD and LLD (Fig. 6-1). Within the cohort of bilateral and left lateralized individuals (*n* = 1,007), it was observed that individuals with left lateralization exhibited higher QA in 2,126 tracts, predominantly in the forceps minor (48%), corpus callosum body (38%), and bilateral fornix (8%), implying that the negative correlations in the primary findings were mainly driven by individuals with bilateral language dominance (*p* < 0.01, FDR corrected). The remaining streamlines consisted of the right IFOF, right AF, and middle cerebellar peduncle (MCP). No tracts were found to be associated with left lateralized individuals. In the analysis encompassing individuals with bilateral and right hemisphere dominance (*n* = 459), only 20 streamlines in the forceps minor exhibited higher QA in individuals with right hemisphere dominance compared with those with bilateral dominance (*p* < 0.01, FDR corrected). No significant differences were observed between LLD and RLD (*n* = 614; *p* < 0.01, FDR corrected).

**Figure 7.**
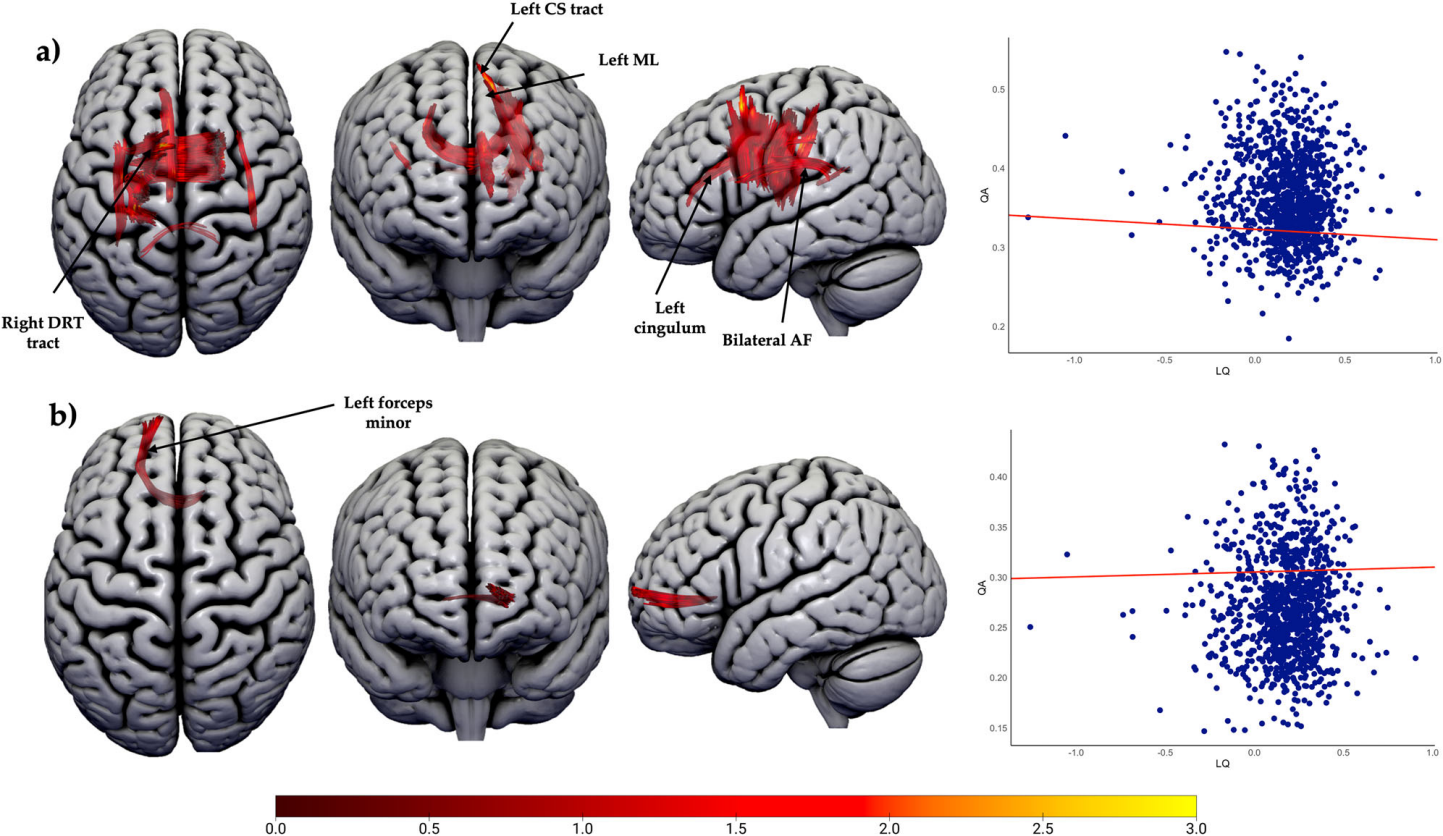
Connectometry results for the language lateralization in the temporal lobe (*n* = 1,040). ***a***, Tract sections negatively correlated with LQ (*p* < 0.01, FDR corrected). ***b***, Tract sections positively correlated with LQ (*p* < 0.01, FDR corrected). Abbreviations: AF, arcuate fasciculus; CC, corpus callosum; CS, corticospinal; DRT, dentatorubrothalamic. The color bar represents the *t*-statistic. See Extended Data Figure 7-1 for the group-level post hoc analyses.

The second connectometry analysis revealed a significantly negative correlation between anterior temporal lobe LQ and QA in 2,408 tracts (*n* = 1,040; *p* < 0.01, FDR corrected; Fig. 7). These tracts were predominantly located in the corpus callosum body (67%), with additional distributions in the left corticospinal tract (9%), left cingulum (7%), left medial lemniscus (5%), right dentarubrothalamic tract (3%), and bilateral AF (3%). Additionally, 118 tracts showed a positive correlation between QA and higher LQ in the anterior temporal regions, all of which were located in the forceps minor (*n* = 1,040; *p* < 0.01, FDR corrected). Categorical post hoc analyses found no significant differences between LLD and BLR (*n* = 1,029; *p* < 0.01, FDR corrected) or between RLD and BLR (*n* = 847; *p* < 0.01, FDR corrected; Fig. 7-1). However, LLD and RLD comparison (*n* = 210) identified 391 streamlines with higher QA in RLD compared with LLD. These streamlines were distributed in the forceps minor (29%), corpus callosum tapetum (27%), bilateral arcuate fasciculus (26%), and right IFOF (8%).

### Shape analysis

**Figure 8.**
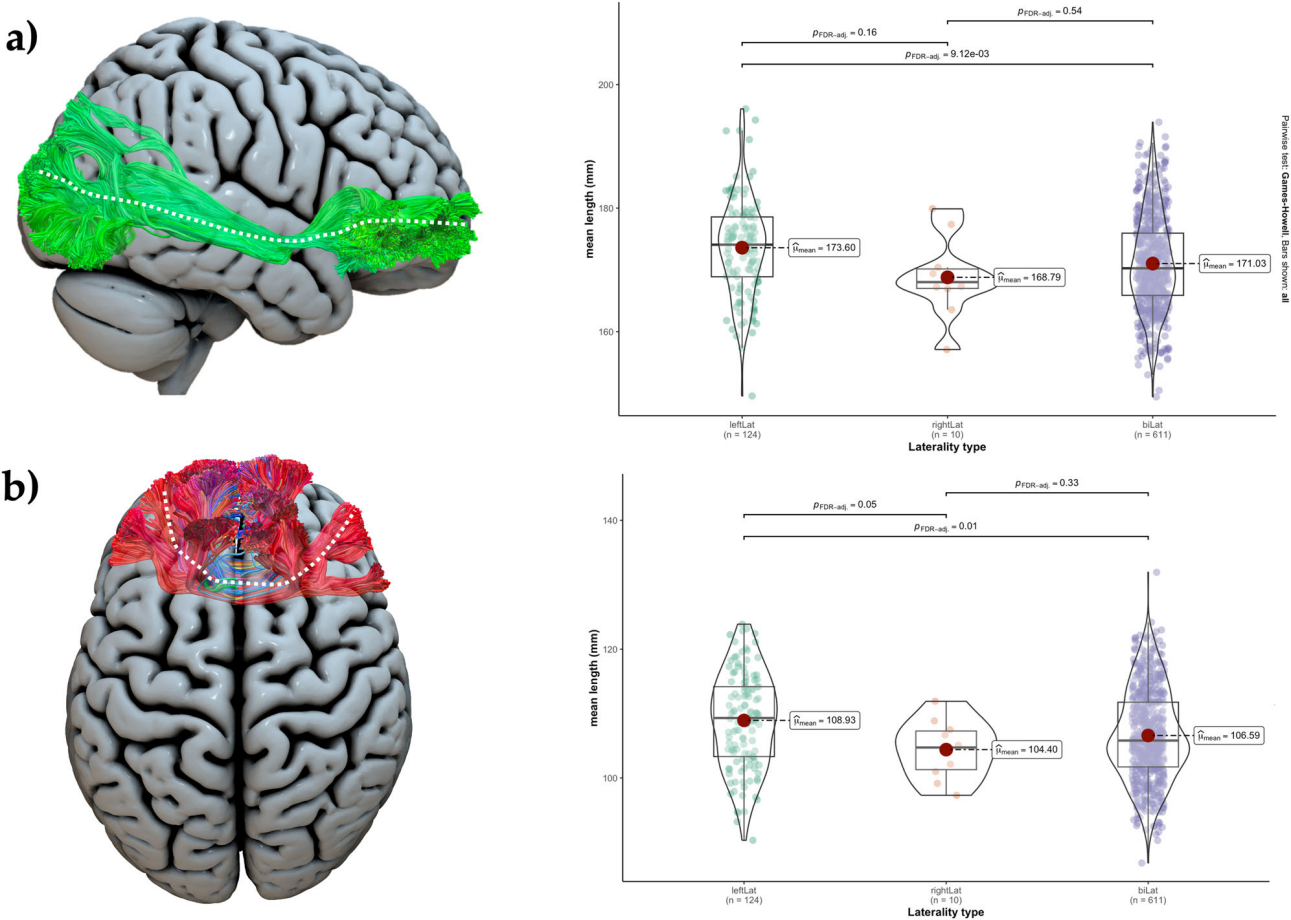
Violin plots (right) illustrate the mean length distribution in (***a***) the right IFOF and (***b***) the forceps minor (illustrated on the left), across different temporal language lateralization groups. Group differences are analyzed using Tukey post hoc tests, with adjustments for FDR. Significant differences are indicated by *p*-values <0.05 (FDR corrected), while nonsignificant differences are denoted by *p*-values >0.05 (FDR corrected).

ANOVA analysis for language lateralization in the anterior temporal lobe showed that the mean length of right IFOF was significantly different between the three laterality groups (*F*_2 _= 9.8; *p* = 0.005, FDR corrected). Tukey post hoc tests showed that left lateralized individuals had longer right IFOF compared with people with BLR (*p* = 0.01, FDR corrected). Another tract that showed a significant difference was forceps minor, with different mean lengths between laterality groups (*F*_2 _= 10.1; *p* = 0.005, FDR corrected). Similarly, Tukey post hoc tests showed that left lateralized people had longer forceps minor compared with bilateral individuals (*p* = 0.01, FDR corrected; Fig. 8). No significant differences between frontal laterality groups were found in relation to shape metrics.

10.1523/JNEUROSCI.0166-24.2024.f6-1 Figure 6-1 Posthoc group connectometry analyses for the language lateralisation in frontal lobe a) Tract sections associated with bilateral language dominance in frontal lobe, when compared to LLD (n = 1007; p < 0.01, FDR corrected). b) Tract sections associated with right lateralisation in frontal lobe when compared to people with BLR (n = 459; p < 0.01, FDR corrected). Abbreviations: AF, arcuate fasciculus; CC, corpus callosum; IFOF – inferior fronto-occipital fasciculus; MCP – middle cerebellar peduncle. Colour bar represents t-statistic. Download Figure 6-1, TIF file.

## Discussion

### Biological implications

We report significantly increased QA of the corpus callosum in individuals who had lower LQ in both frontal and temporal ROIs. Post hoc analyses revealed that this result was driven by individuals with both BLR and RLD when the laterality quotient was calculated using frontal regions and by RLD when the laterality quotient was based on the anterior temporal lobe. This observation aligns with prior research in patient cohorts (Tantillo et al., 2016) and supports the hypothesis that individuals who rely more on both cerebral hemispheres for language processing may have more developed commissural fibers. These fibers could potentially serve as a compensatory mechanism for the heightened metabolic energy requirements associated with information transfer and the reduction of transmission times (Laughlin and Sejnowski, 2003). An alternative hypothesis suggests that using both hemispheres for language processing is more natural than previously thought (Newport et al., 2022). This implies that the shift of language function toward the left hemisphere might result from less developed commissural tracts. Well-developed commissural tracts may facilitate bilateral language processing in frontal regions by allowing the right hemisphere to participate fully in language tasks (Newport et al., 2017). A higher forceps minor anisotropy in right lateralized people compared with people with LLD and BLR (Extended Data Figs. 6-1, 7-1) suggests that commissural tracts may not necessarily enhance the function of both hemispheres but rather maintain right hemisphere involvement in language comprehension tasks.

Other imaging research has also suggested the importance of the corpus callosum for language lateralization, although the link between structural measures of the corpus callosum and HLD is unclear. One study reported a greater FA of the whole corpus callosum in people with atypical language lateralization (defined as RLD and BLR together) and, consistent with our results of anterior temporal lobe lateralization, greatest anisotropy in people with RLD (Häberling et al., 2011). We found a positive correlation between a small segment of the left forceps minor and temporal LQ, suggesting a more complex role of the forceps minor than previously understood (Fig. 7). This finding was supported by shape analysis, which revealed a longer forceps minor in individuals with left temporal language lateralization. This aligns with a recent study using fixel-based analysis, which reported greater left fiber bundle cross-sectional asymmetry of the forceps minor in individuals with left language dominance (LLD) and higher asymmetry of the right forceps minor in those with RLD (Verhelst et al., 2021). Thus, while the body of the corpus callosum is associated with atypical (both bilateral and right) lateralization for language comprehension in our study, the posterior and anterior parts of the corpus callosum are mainly associated with lateralized language, whether right or left, consistent with previous studies (Westerhausen et al., 2006; Karpychev et al., 2022). Although commissural fibers have shown relationships with language lateralization in both previous and current studies, there is inconsistency regarding which individuals exhibit greater microstructural differences inferred by diffusion scalar metrics. This inconsistency may stem from methodological factors, such as the inclusion or exclusion of individuals with BLR and the type of language lateralization assessed.

10.1523/JNEUROSCI.0166-24.2024.f7-1 Figure 7-1 Posthoc group connectometry analyses for the language lateralisation in temporal lobe. Right lateralised people were associated with commissural tracts and bilateral AF (n = 207; p < 0.01, FDR corrected). Abbreviations: AF, arcuate fasciculus; CC, corpus callosum. Download Figure 7-1, TIF file.

**Table 1.** A list of abbreviations used in this article

Abbreviation	Definition
AF	Arcuate fasciculus
ANOVA	Analysis of variance
BLR	Bilateral language representation
BOLD	Blood oxygenation level–dependent signal
CIFTI	Connectivity Informatics Technology Initiative
COPE	Contrast of parameter estimates (COPE) map
FDR	False discovery rate
FAT	Frontal aslant tract
FNIRT	A “medium resolution” nonlinear registration method
FSL	The FMRIB Software Library
FWE	Family-wise error
fMRI	Functional magnetic resonance imaging
HCP	Human Connectome Project
HLD	Hemispheric language dominance
IFOF	Inferior fronto-occipital fasciculus
ILF	Inferior longitudinal fasciculus
LLD	Left language dominance
LQ	Laterality quotient
MNI	Montreal Neurological Institute
MSM-ALL	Multimodal Surface Matching registration
MSM-SULC	Cortical folding-based registration
QA	Quantitative anisotropy
ROI	Region of interest
RLD	Right language dominance
SDF	Spin distribution function
SLF	Superior longitudinal fasciculus
STGa	Superior temporal gyrus anterior
STSda	Superior temporal sulcus dorsal anterior
STSva	Superior temporal sulcus ventral anterior
TE1a	Temporal Area 1 anterior
TE2a	Temporal Area 2 anterior
TGd	Temporal gyrus dorsal
TGv	Temporal gyrus ventral

Our study reported a significant association between asymmetry in frontal activation and QA in the fornix bilaterally, particularly in individuals exhibiting an LQ closer to zero, indicating a more bilateral hemispheric representation. While there is limited existing literature on the relationship between HLD and diffusion measures in these tracts, some have highlighted the involvement of them in language processing (Hula et al., 2020; Sihvonen et al., 2021). Our findings related to temporal lobe laterality revealed a small number of streamlines in the AF bilaterally exhibiting higher anisotropy in individuals with RLD compared with LLD, but not in those with BLR compared with LLD. This suggests that increased QA in these regions may be linked to right hemisphere language lateralization. Previous research has also found associations between AF asymmetry and language lateralization in several cohorts of healthy individuals (Propper et al., 2010; Ocklenburg et al., 2013), although the exact relationship between AF asymmetry and HLD remains unclear (Verhelst et al., 2021; Gerrits et al., 2022). Further studies are needed to better understand these tracts’ importance for language lateralization.

### Methodological considerations

To investigate the relationship between language lateralization and white matter characteristics, we used both correlational methods and traditional categorical classifications (left, right, bilateral). Analyzing raw LQ values for the entire sample helped reduce the subjectivity inherent in categorical groupings (Westerhausen et al., 2006; Wegrzyn et al., 2019). Instead of relying solely on global BOLD activation patterns to assess language lateralization, we conducted two distinct studies focusing on the frontal and anterior temporal lobes. This approach allowed us to obtain more detailed insights. For instance, while lower LQs in both frontal and temporal regions were associated with increased anisotropy in the corpus callosum, frontal regions engaged a larger portion of the anterior corpus callosum body and the forceps minor. Interestingly, lower LQs in the temporal lobe were linked to increased anisotropy in the bilateral AF, which suggested a right hemisphere dominance, as indicated by post hoc analyses—an effect not observed in the frontal lobe. Furthermore, while frontal LQs were positively correlated with fractional anisotropy in the bilateral cingulum, temporal LQs were associated with increased QA in the left forceps minor. Analyzing language lateralization separately for the frontal and temporal regions thus provided richer and more nuanced insights into the relationship between language comprehension and white matter characteristics.

The number of individuals with right hemisphere dominance, especially for the anterior temporal region, is small in the present study compared with previous studies (Chang et al., 2011). This disparity is primarily attributed to the task used to assess HLD, which entailed semantic processing, resulting in more bilateral fMRI activations (Binder et al., 2011; Walenski et al., 2019; Metoki et al., 2022). This might be because comprehension tasks rely less on the left-lateralised dorsal pathway, strongly associated with cognitive operations crucial for language production (e.g., retrieval and production of speech sounds; Hickok and Poeppel 2007). Other nonmutually exclusive explanations include differences in the complexity of the linguistic stimuli (single words typically used in fluency tasks versus sentences typically used in comprehension tasks) and the demands that these tasks typically involve (Peelle, 2012). For example, the verbal stimuli used for the stories likely portray social concepts (e.g., theory of mind, intention, emotion, morality), and/or they may contain different amounts of metaphor, idiom, or implied meaning. All these aspects have been associated with the recruitment of frontotemporal and parietal regions in the right hemisphere (Miller et al., 1997; Olson et al., 2007; Schmidt et al., 2007; Yang, 2014).

The fact that we employed a language comprehension task might explain why only a small subset of participants demonstrated strong leftward lateralization, while the majority displayed mild lateralization. A recent systematic review comparing different language tasks has highlighted that language production tasks may be more robust in accurately assessing language laterality than language comprehension tasks (Bradshaw et al., 2017). For instance, there is evidence that between 6 and 24 years of age, there is an increase in frontal asymmetry during tasks involving the articulation of words. However, this asymmetry is not present during story listening. This suggests partly different maturational mechanisms between language comprehension and production (Berl et al., 2010; Lidzba et al., 2011). Future studies will have to use tasks that typically generate strongly left lateralized neural responses such as verbal fluency tasks to corroborate our findings.

Our methodology offers several practical benefits. Firstly, despite the limited proportion of right hemisphere dominant individuals identified through the functional language task, the sizeable sample size (*N* = 1,040) employed in this study is the largest ever utilized in such investigations, thus potentially decreasing the likelihood of false positives. Secondly, we employed two complementary methodologies, namely connectometry and tractography, to delineate the precise white matter characteristics associated with HLD. In doing so, we have advanced our understanding of the anatomical basis of language lateralisation, demonstrating that white matter tract microstructural properties (QA) exhibit stronger associations with HLD compared to geometrical features (shape analysis).

## Conclusion

The findings of our study suggest that measures of the diffusion-based microstructural architecture of reconstructed white matter tracts are linked to language lateralization. Specifically, individuals who exhibit a greater reliance on both cerebral hemispheres for language comprehension may possess more highly developed CC body fibers, thereby promoting more efficient interhemispheric communication. The involvement of anterior and posterior parts of the CC in asymmetrical temporal lobe activity (i.e., either left or right) for language comprehension unveils a more complex and nuanced role of the forceps minor. Future research should further investigate these relationships, employing tasks that typically generate strongly left lateralized neural responses, to validate and expand upon our findings.

## References

[B1] Andersson JL, Sotiropoulos SN (2016) An integrated approach to correction for off-resonance effects and subject movement in diffusion MR imaging. Neuroimage 125:1063–1078. 10.1016/j.neuroimage.2015.10.019 26481672 PMC4692656

[B2] Ashraf-Ganjouei A, Rahmani F, Aarabi MH, Sanjari Moghaddam H, Nazem-Zadeh MR, Davoodi-Bojd E, Soltanian-Zadeh H (2019) White matter correlates of disease duration in patients with temporal lobe epilepsy: updated review of literature. Neurol Sci 40:1209–1216. 10.1007/s10072-019-03818-2 30868482

[B3] Baldo JV, Dronkers NF (2007) Neural correlates of arithmetic and language comprehension: a common substrate? Neuropsychologia 45:229–235. 10.1016/j.neuropsychologia.2006.07.014 16997333

[B4] Barba C, et al. (2020) Patterns and predictors of language representation and the influence of epilepsy surgery on language reorganization in children and young adults with focal lesional epilepsy. PLoS One 15:e0238389. 10.1371/journal.pone.0238389 32898166 PMC7478845

[B5] Barnes-Davis ME, Williamson BJ, Merhar SL, Holland SK, Kadis DS (2020) Extremely preterm children exhibit altered cortical thickness in language areas. Sci Rep 10:10824. 10.1038/s41598-020-67662-7 32616747 PMC7331674

[B6] Barnes-Davis ME, Williamson BJ, Merhar SL, Nagaraj UD, Parikh NA, Kadis DS (2022) Extracallosal structural connectivity is positively associated with language performance in well-performing children born extremely preterm. Front Pediatr 10:821121. 10.3389/fped.2022.821121 35372163 PMC8971711

[B7] Berl MM, Duke ES, Mayo J, Rosenberger LR, Moore EN, VanMeter J, Ratner NB, Vaidya CJ, Gaillard WD (2010) Functional anatomy of listening and reading comprehension during development. Brain Lang 114:115–125. 10.1016/j.bandl.2010.06.002 20656105 PMC2962416

[B8] Binder JR, et al. (2011) Mapping anterior temporal lobe language areas with fMRI: a multicenter normative study. Neuroimage 54:1465–1475. 10.1016/j.neuroimage.2010.09.048 20884358 PMC2997157

[B9] Bradshaw AR, Thompson PA, Wilson AC, Bishop DV, Woodhead ZV (2017) Measuring language lateralisation with different language tasks: a systematic review. PeerJ 5:e3929. 10.7717/peerj.3929 29085748 PMC5659218

[B10] Branzi FM, Humphreys GF, Hoffman P, Ralph MAL (2020) Revealing the neural networks that extract conceptual gestalts from continuously evolving or changing semantic contexts. Neuroimage 220:116802. 10.1016/j.neuroimage.2020.116802 32283276 PMC7573538

[B11] Branzi FM, Pobric G, Jung J, Lambon Ralph MA (2021) The left angular gyrus is causally involved in context-dependent integration and associative encoding during narrative reading. J Cogn Neurosci 33:1082–1095. 10.1162/jocn_a_01698 34428784 PMC7614446

[B12] Catani M, Allin MP, Husain M, Pugliese L, Mesulam MM, Murray RM, Jones DK (2007) Symmetries in human brain language pathways correlate with verbal recall. Proc Natl Acad Sci U S A 104:17163–17168. 10.1073/pnas.0702116104 17939998 PMC2040413

[B13] Chang EF, Wang DD, Perry DW, Barbaro NM, Berger MS (2011) Homotopic organization of essential language sites in right and bilateral cerebral hemispheric dominance. J Neurosurg 114:893–902. 10.3171/2010.11.JNS10888 21235314

[B14] Chiarello C, Vazquez D, Felton A, Leonard CM (2013) Structural asymmetry of anterior insula: behavioral correlates and individual differences. Brain Lang 126:109–122. 10.1016/j.bandl.2013.03.005 23681069 PMC3722256

[B15] Dietz A, Vannest J, Maloney T, Altaye M, Szaflarski JP, Holland SK (2016) The calculation of language lateralization indices in post-stroke aphasia: a comparison of a standard and a lesion-adjusted formula. Front Hum Neurosci 10:493. 10.3389/fnhum.2016.00493 27790102 PMC5061744

[B16] Dorsaint-Pierre R, Penhune VB, Watkins KE, Neelin P, Lerch JP, Bouffard M, Zatorre RJ (2006) Asymmetries of the planum temporale and heschl's gyrus: relationship to language lateralisation. Brain 129:1164–1176. 10.1093/brain/awl055 16537567

[B17] Dresang HC, Hula WD, Yeh FC, Warren T, Dickey MW (2021) White-matter neuroanatomical predictors of aphasic verb retrieval. Brain Connect 11:319–330. 10.1089/brain.2020.0921 33470167 PMC8112714

[B18] Elam JS, et al. (2021) The human connectome project: a retrospective. Neuroimage 244:118543. 10.1016/j.neuroimage.2021.118543 34508893 PMC9387634

[B19] Fischl B (2012) Freesurfer. Neuroimage 62:774–781. 10.1016/j.neuroimage.2012.01.021 22248573 PMC3685476

[B20] Fischl B, Rajendran N, Busa E, Augustinack J, Hinds O, Yeo BT, Mohlberg H, Amunts K, Zilles K (2008) Cortical folding patterns and predicting cytoarchitecture. Cerebral cortex 18:1973–1980. 10.1093/cercor/bhm225 18079129 PMC2474454

[B21] Forkel SJ, Friedrich P, Thiebaut de Schotten M, Howells H (2022) White matter variability, cognition, and disorders: a systematic review. Brain Struct Funct 227:529–544. 10.1007/s00429-021-02382-w 34731328 PMC8844174

[B22] Foundas AL, Leonard CM, Gilmore RL, Fennell EB, Heilman KM (1996) Pars triangularis asymmetry and language dominance. Proc Natl Acad Sci U S A 93:719–722. 10.1073/pnas.93.2.719 8570622 PMC40120

[B23] Friederici AD, von Cramon DY, Kotz SA (2007) Role of the corpus callosum in speech comprehension: interfacing syntax and prosody. Neuron 53:135–145. 10.1016/j.neuron.2006.11.020 17196536

[B24] Gerrits R, Verhelst H, Dhollander T, Xiang L, Vingerhoets G (2022) Structural perisylvian asymmetry in naturally occurring atypical language dominance. Brain Struct Funct 227:573–586. 10.1007/s00429-021-02323-7 34173870

[B25] Glasser MF, et al. (2013) The minimal preprocessing pipelines for the human connectome project. Neuroimage 80:105–124. 10.1016/j.neuroimage.2013.04.127 23668970 PMC3720813

[B26] Glasser MF, et al. (2016) A multi-modal parcellation of human cerebral cortex. Nature 536:171–178. 10.1038/nature18933 27437579 PMC4990127

[B27] Greve DN, Van der Haegen L, Cai Q, Stufflebeam S, Sabuncu MR, Fischl B, Brysbaert M (2013) A surface-based analysis of language lateralisation and cortical asymmetry. J Cogn Neurosci 25:1477–1492. 10.1162/jocn_a_00405 23701459 PMC3767398

[B28] Güntürkün O, Ströckens F, Ocklenburg S (2020) Brain lateralization: a comparative perspective. Physiol Rev 100:1019–1063. 10.1152/physrev.00006.2019 32233912

[B29] Häberling IS, Badzakova-Trajkov G, Corballis MC (2011) Callosal tracts and patterns of hemispheric dominance: a combined fMRI and DTI study. Neuroimage 54:779–786. 10.1016/j.neuroimage.2010.09.072 20920586

[B30] Harvey DY, Wei T, Ellmore TM, Hamilton AC, Schnur TT (2013) Neuropsychological evidence for the functional role of the uncinate fasciculus in semantic control. Neuropsychologia 51:789–801. 10.1016/j.neuropsychologia.2013.01.028 23395830

[B31] Hickok G, Poeppel D (2007) The cortical organization of speech processing. Nat Rev Neurosci 8:393–402. 10.1038/nrn2113 17431404

[B32] Hula WD, Panesar S, Gravier ML, Yeh FC, Dresang HC, Dickey MW, Fernandez-Miranda JC (2020) Structural white matter connectometry of word production in aphasia: an observational study. Brain 143:2532–2544. 10.1093/brain/awaa193 32705146 PMC7447522

[B33] Humphreys GF, Lambon Ralph MA (2015) Fusion and fission of cognitive functions in the human parietal cortex. Cereb Cortex 25:3547–3560. 10.1093/cercor/bhu198 25205661 PMC4585503

[B34] Ivanova MV, Zhong A, Turken A, Baldo JV, Dronkers NF (2021) Functional contributions of the arcuate fasciculus to language processing. Front Hum Neurosci 15:672665. 10.3389/fnhum.2021.672665 34248526 PMC8267805

[B35] Jackson RL (2021) The neural correlates of semantic control revisited. Neuroimage 224:117444. 10.1016/j.neuroimage.2020.117444 33059049 PMC7779562

[B36] James JS, Kumari SR, Sreedharan RM, Thomas B, Radhkrishnan A, Kesavadas C (2015) Analyzing functional, structural, and anatomical correlation of hemispheric language lateralisation in healthy subjects using functional MRI, diffusion tensor imaging, and voxel-based morphometry. Neurol India 63:49. 10.4103/0028-3886.152634 25751469

[B37] Jenkinson M, Beckmann CF, Behrens TE, Woolrich MW, Smith SM (2012) Fsl. Neuroimage 62:782–790. 10.1016/j.neuroimage.2011.09.015 21979382

[B38] Josse G, Kherif F, Flandin G, Seghier ML, Price CJ (2009) Predicting language lateralisation from gray matter. J Neurosci 29:13516–13523. 10.1523/JNEUROSCI.1680-09.2009 19864564 PMC2795346

[B39] Karpychev V, Bolgina T, Malytina S, Zinchenko V, Ushakov V, Ignatyev G, Dragoy O (2022) Greater volumes of a callosal sub-region terminating in posterior language-related areas predict a stronger degree of language lateralisation: a tractography study. PLoS One 17:e0276721. 10.1371/journal.pone.0276721 36520829 PMC9754228

[B40] Keller SS, Roberts N, Baker G, Sluming V, Cezayirli E, Mayes A, Eldridge P, Marson AG, Wieshmann UC (2018) A voxel-based asymmetry study of the relationship between hemispheric asymmetry and language dominance in Wada tested patients. Hum Brain Mapp 39:3032–3045. 10.1002/hbm.24058 29569808 PMC6055618

[B41] Keller SS, Roberts N, García-Fiñana M, Mohammadi S, Ringelstein EB, Knecht S, Deppe M (2011) Can the language-dominant hemisphere be predicted by brain anatomy? J Cogn Neurosci 23:2013–2029. 10.1162/jocn.2010.21563 20807056

[B42] Laughlin SB, Sejnowski TJ (2003) Communication in neuronal networks. Science 301:1870–1874. 10.1126/science.1089662 14512617 PMC2930149

[B43] Lerner Y, Honey CJ, Silbert LJ, Hasson U (2011) Topographic mapping of a hierarchy of temporal receptive windows using a narrated story. J Neurosci 31:2906–2915. 10.1523/JNEUROSCI.3684-10.2011 21414912 PMC3089381

[B44] Lidzba K, Schwilling E, Grodd W, Krägeloh-Mann I, Wilke M (2011) Language comprehension versus language production: age effects on fMRI activation. Brain Lang 119:6–15. 10.1016/j.bandl.2011.02.003 21450336

[B45] Marcus DS, et al. (2013) Human connectome project informatics: quality control, database services, and data visualization. Neuroimage 80:202–219. 10.1016/j.neuroimage.2013.05.077 23707591 PMC3845379

[B46] Metoki A, Wang Y, Olson IR (2022) The social cerebellum: a large-scale investigation of functional and structural specificity and connectivity. Cereb Cortex 32:987–1003. 10.1093/cercor/bhab260 34428293 PMC8890001

[B47] Miller BL, Darby A, Benson DF, Cummings JL, Miller MH (1997) Aggressive, socially disruptive and antisocial behaviour associated with fronto-temporal dementia. The Br J Psychiatry 170:150–154. 10.1192/bjp.170.2.150 9093504

[B48] Newport EL, et al. (2022) Language and developmental plasticity after perinatal stroke. Proc Natl Acad Sci U S A 119:e2207293119. 10.1073/pnas.2207293119 36215488 PMC9586296

[B49] Newport EL, Landau B, Seydell-Greenwald A, Turkeltaub PE, Chambers CE, Dromerick AW, Carpenter J, Berl MM, Gaillard WD (2017) Revisiting Lenneberg’s hypotheses about early developmental plasticity: language organization after left-hemisphere perinatal stroke. Biolinguistics 11:407. 10.5964/bioling.9105 30556058 PMC6291004

[B50] Ocklenburg S, Hugdahl K, Westerhausen R (2013) Structural white matter asymmetries in relation to functional asymmetries during speech perception and production. Neuroimage 83:1088–1097. 10.1016/j.neuroimage.2013.07.076 23921095

[B51] Oldfield RC (1971) The assessment and analysis of handedness: the Edinburgh inventory. Neuropsychologia 9:97–113. 10.1016/0028-3932(71)90067-4 5146491

[B52] Olson IR, Plotzker A, Ezzyat Y (2007) The enigmatic temporal pole: a review of findings on social and emotional processing. Brain 130:1718–1731. 10.1093/brain/awm052 17392317

[B53] Peelle JE (2012) The hemispheric lateralization of speech processing depends on what “speech” is: a hierarchical perspective. Front Hum Neurosci 6:309. 10.3389/fnhum.2012.00309 23162455 PMC3499798

[B54] Perlaki G, et al. (2013) White-matter microstructure and language lateralisation in left-handers: a whole-brain MRI analysis. Brain Cogn 82:319–328. 10.1016/j.bandc.2013.05.005 23792788

[B55] Pham DD, Muschelli J, Mejia AF (2022) Ciftitools: a package for reading, writing, visualizing, and manipulating CIFTI files in R. Neuroimage 250:118877. 10.1016/j.neuroimage.2022.118877 35051581 PMC9119143

[B56] Powell HR, Parker GJ, Alexander DC, Symms MR, Boulby PA, Wheeler-Kingshott CA, Barker GJ, Noppeney U, Koepp MJ, Duncan JS (2006) Hemispheric asymmetries in language-related pathways: a combined functional MRI and tractography study. Neuroimage 32:388–399. 10.1016/j.neuroimage.2006.03.011 16632380

[B57] Propper RE, O’Donnell LJ, Whalen S, Tie Y, Norton IH, Suarez RO, Zollei L, Radmanesh A, Golby AJ (2010) A combined fMRI and DTI examination of functional language lateralisation and arcuate fasciculus structure: effects of degree versus direction of hand preference. Brain Cogn 73:85–92. 10.1016/j.bandc.2010.03.004 20378231 PMC2880216

[B58] Rahmani F, Sobhani S, Aarabi MH (2017) Sequential language learning and language immersion in bilingualism: diffusion MRI connectometry reveals microstructural evidence. Exp Brain Res 235:2935–2945. 10.1007/s00221-017-5029-x 28702836

[B59] Robinson EC, et al. (2018) Multimodal surface matching with higher-order smoothness constraints. Neuroimage 167:453–465. 10.1016/j.neuroimage.2017.10.037 29100940 PMC5991912

[B60] Rollans C, Cummine J (2018) One tract, two tract, old tract, new tract: a pilot study of the structural and functional differentiation of the inferior fronto-occipital fasciculus. J Neurolinguistics 46:122–137. 10.1016/j.jneuroling.2017.12.009

[B61] Sabbah P, et al. (2003) Functional MR imaging in assessment of language dominance in epileptic patients. Neuroimage 18:460–467. 10.1016/S1053-8119(03)00025-9 12595199

[B62] Schilling KG, Yeh FC, Nath V, Hansen C, Williams O, Resnick S, Anderson AW, Landman BA (2019) A fiber coherence index for quality control of B-table orientation in diffusion MRI scans. Magn Reson Imaging 58:82–89. 10.1016/j.mri.2019.01.018 30682379 PMC6401245

[B63] Schmidt GL, DeBuse CJ, Seger CA (2007) Right hemisphere metaphor processing? Characterizing the lateralization of semantic processes. Brain and Lang 100:127–141. 10.1016/j.bandl.2005.03.002 17292739

[B64] Seghier ML (2008) Laterality index in functional MRI: methodological issues. Magn Reson Imaging 26:594–601. 10.1016/j.mri.2007.10.010 18158224 PMC2726301

[B65] Seghier ML (2019) Categorical laterality indices in fMRI: a parallel with classic similarity indices. Brain Struct Funct 224:1377–1383. 10.1007/s00429-019-01833-9 30656446

[B66] Seghier ML, Lazeyras F, Pegna AJ, Annoni JM, Khateb A (2008) Group analysis and the subject factor in functional magnetic resonance imaging: analysis of fifty right-handed healthy subjects in a semantic language task. Hum Brain Mapp 29:461–477. 10.1002/hbm.20410 17538950 PMC6870607

[B67] Shin J, Rowley J, Chowdhury R, Jolicoeur P, Klein D, Grova C, Rosa-Neto P, Kobayashi E (2019) Inferior longitudinal fasciculus’ role in visual processing and language comprehension: a combined MEG-DTI study. Front Neurosci 13:875. 10.3389/fnins.2019.00875 31507359 PMC6716060

[B68] Sihvonen AJ, Virtala P, Thiede A, Laasonen M, Kujala T (2021) Structural white matter connectometry of reading and dyslexia. Neuroimage 241:118411. 10.1016/j.neuroimage.2021.118411 34293464

[B69] Silva G, Citterio A (2017) Hemispheric asymmetries in dorsal language pathway white-matter tracts: a magnetic resonance imaging tractography and functional magnetic resonance imaging study. Neuroradiol J 30:470–476. 10.1177/1971400917720829 28699372 PMC5602342

[B70] Sotiropoulos SN, et al. (2013) Advances in diffusion MRI acquisition and processing in the Human Connectome Project. Neuroimage 80:125–143. 10.1016/j.neuroimage.2013.05.057 23702418 PMC3720790

[B71] Spitsyna G, Warren JE, Scott SK, Turkheimer FE, Wise RJ (2006) Converging language streams in the human temporal lobe. J Neurosci 26:7328–7336. 10.1523/JNEUROSCI.0559-06.2006 16837579 PMC6674192

[B72] Tantillo G, Peck KK, Arevalo-Perez J, Lyo JK, Chou JF, Young RJ, Brennan NP, Holodny AI (2016) Corpus callosum diffusion and language lateralisation in patients with brain tumors: a DTI and fMRI study. J Neuroimaging 26:224–231. 10.1111/jon.12275 26258653 PMC4753142

[B73] Timocin G, Toprak A, Aralasmak A (2020) Relationships of language lateralisation with diffusion tensor imaging metrics of corpus callosum, tumor grade, and tumors distance to language-eloquent areas in glial neoplasms. J Comput Assist Tomogr 44:956–968. 10.1097/RCT.0000000000001103 33196603

[B74] Van Essen DC (2005) A population-average, landmark-and surface-based (PALS) atlas of human cerebral cortex. Neuroimage 28:635–662. 10.1016/j.neuroimage.2005.06.058 16172003

[B75] Van Essen DC, Glasser MF (2016) The Human Connectome Project: progress and prospects. Cerebrum 10–16.

[B76] Van Essen DC, Smith SM, Barch DM, Behrens TE, Yacoub E, Ugurbil K, Wu-Minn HCP Consortium (2013) The WU-minn human connectome project: an overview. Neuroimage 80:62–79. 10.1016/j.neuroimage.2013.05.041 23684880 PMC3724347

[B77] Verhelst H, Dhollander T, Gerrits R, Vingerhoets G (2021) Fibre-specific laterality of white matter in left and right language dominant people. Neuroimage 230:117812. 10.1016/j.neuroimage.2021.117812 33524578

[B78] Vernooij MW, Smits M, Wielopolski PA, Houston GC, Krestin GP, van der Lugt A (2007) Fiber density asymmetry of the arcuate fasciculus in relation to functional hemispheric language lateralisation in both right-and left-handed healthy subjects: a combined fMRI and DTI study. Neuroimage 35:1064–1076. 10.1016/j.neuroimage.2006.12.041 17320414

[B79] Vingerhoets G, et al. (2023) Laterality indices consensus initiative (LICI): a Delphi expert survey report on recommendations to record, assess, and report asymmetry in human behavioural and brain research. Laterality 28:122–191. 10.1080/1357650X.2023.2199963 37211653

[B80] Walenski M, Europa E, Caplan D, Thompson CK (2019) Neural networks for sentence comprehension and production: an ALE-based meta-analysis of neuroimaging studies. Hum Brain Mapp 40:2275–2304. 10.1002/hbm.24523 30689268 PMC6597252

[B81] Wegrzyn M, Mertens M, Bien CG, Woermann FG, Labudda K (2019) Quantifying the confidence in fMRI-based language lateralisation through laterality index deconstruction. Front Neurol 10:655. 10.3389/fneur.2019.00655 31275236 PMC6594217

[B82] Westerhausen R, Kreuder F, Sequeira SDS, Walter C, Woerner W, Wittling RA, Schweiger E, Wittling W (2006) The association of macro-and microstructure of the corpus callosum and language lateralisation. Brain Lang 97:80–90. 10.1016/j.bandl.2005.07.133 16157367

[B83] Yang J (2014) The role of the right hemisphere in metaphor comprehension: a meta-analysis of functional magnetic resonance imaging studies. Hum Brain Mapp 35:107–122. 10.1002/hbm.22160 22936560 PMC6868953

[B84] Yeh FC (2020) Shape analysis of the human association pathways. Neuroimage 223:117329. 10.1016/j.neuroimage.2020.117329 32882375 PMC7775618

[B85] Yeh FC, Badre D, Verstynen T (2016) Connectometry: a statistical approach harnessing the analytical potential of the local connectome. Neuroimage 125:162–171. 10.1016/j.neuroimage.2015.10.053 26499808

[B86] Yeh FC, Panesar S, Barrios J, Fernandes D, Abhinav K, Meola A, Fernandez-Miranda JC (2019) Automatic removal of false connections in diffusion MRI tractography using topology-informed pruning (TIP). Neurotherapeutics 16:52–58. 10.1007/s13311-018-0663-y 30218214 PMC6361061

[B87] Yeh FC, Panesar S, Fernandes D, Meola A, Yoshino M, Fernandez-Miranda JC, Vettel JM, Verstynen T (2018) Population-averaged atlas of the macroscale human structural connectome and its network topology. Neuroimage 178:57–68. 10.1016/j.neuroimage.2018.05.027 29758339 PMC6921501

[B88] Yeh FC, Tseng WYI (2011) NTU-90: a high angular resolution brain atlas constructed by q-space diffeomorphic reconstruction. Neuroimage 58:91–99. 10.1016/j.neuroimage.2011.06.021 21704171

[B89] Yeh FC, Verstynen TD, Wang Y, Fernández-Miranda JC, Tseng WYI (2013) Deterministic diffusion fiber tracking improved by quantitative anisotropy. PLoS One 8:e80713. 10.1371/journal.pone.0080713 24348913 PMC3858183

[B90] Yeh FC, Wedeen VJ, Tseng WYI (2010) Generalized *q*-sampling imaging. IEEE Trans Med Imaging 29:1626–1635. 10.1109/TMI.2010.2045126 20304721

[B91] Zhong AJ, Baldo JV, Dronkers NF, Ivanova MV (2022) The unique role of the frontal aslant tract in speech and language processing. Neuroimage Clin 34:103020. 10.1016/j.nicl.2022.103020 35526498 PMC9095886

