# Supplementary figures and images for: The Relationship between White Matter Architecture and Language Lateralization in the Healthy Brain
Source: J Neurosci. 2024 Oct 7;44(50):e0166242024. doi: 10.1523/JNEUROSCI.0166-24.2024 (PMC11638810; doi:10.1523/JNEUROSCI.0166-24.2024)

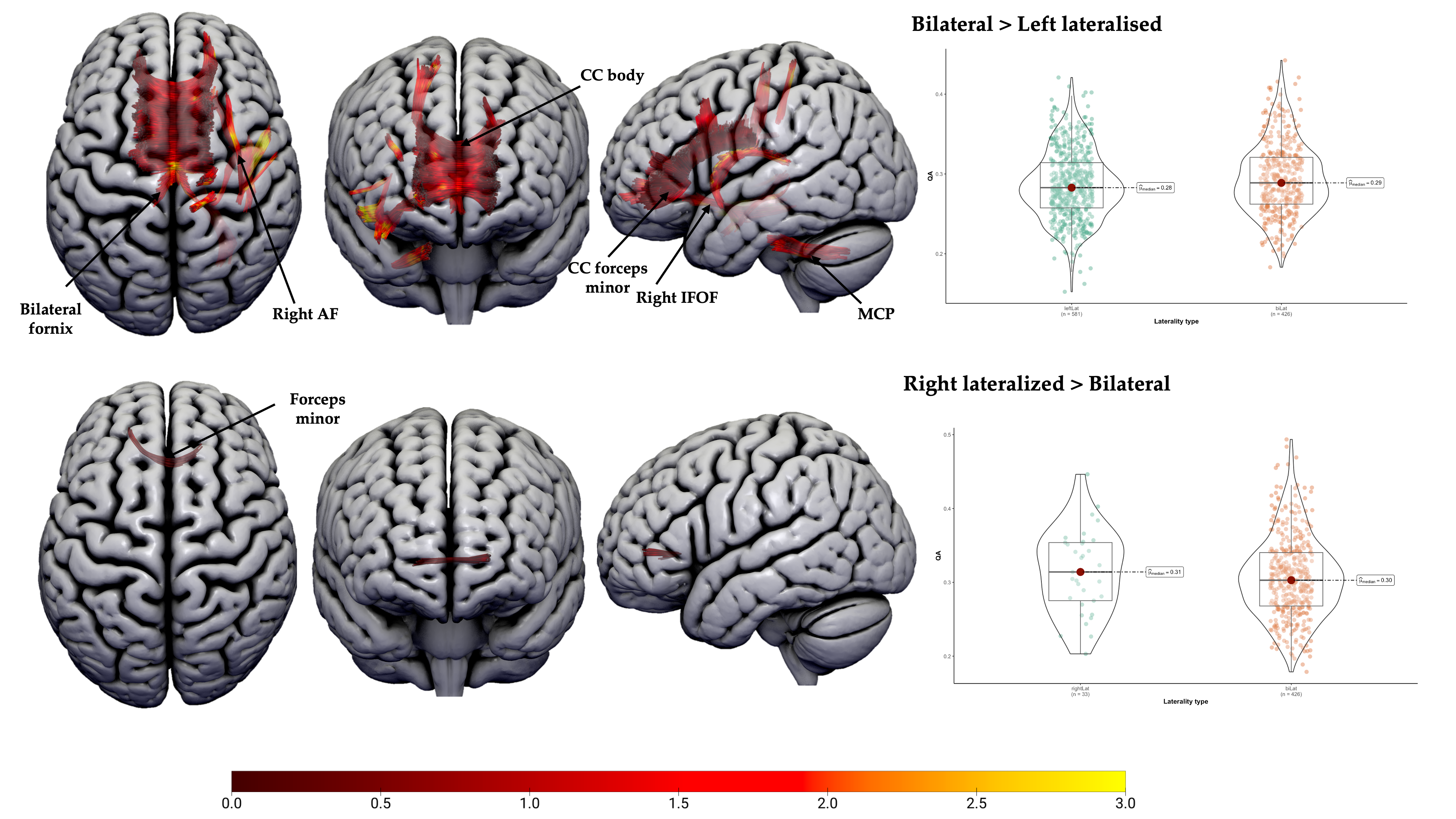

Supplement: Figure 6-1 — Posthoc group connectometry analyses for the language lateralisation in frontal lobe a) Tract sections associated with bilateral language dominance in frontal lobe, when compared to LLD (n = 1007; p < 0.01, FDR corrected). b) Tract sections associated with right lateralisation in frontal lobe when compared to people with BLR (n = 459; p < 0.01, FDR corrected). Abbreviations: AF, arcuate fasciculus; CC, corpus callosum; IFOF – inferior fronto-occipital fasciculus; MCP – middle cerebellar peduncle. Colour bar represents t-statistic. Download Figure 6-1, TIF file. [file jneuro-44-e0166242024-s001.tif]

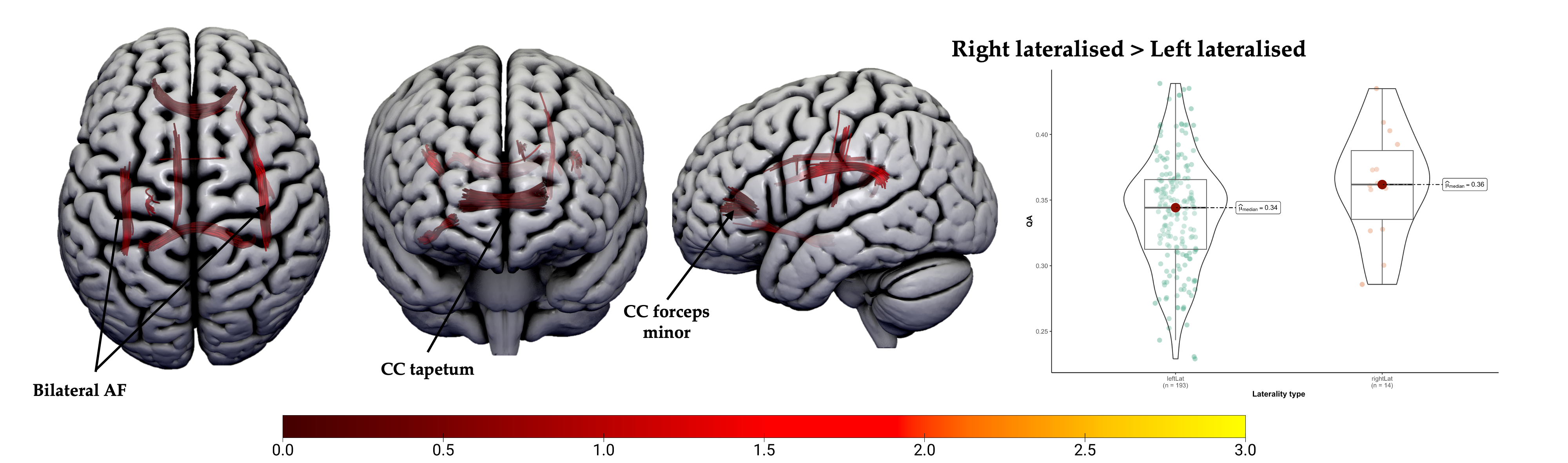

Supplement: Figure 7-1 — Posthoc group connectometry analyses for the language lateralisation in temporal lobe. Right lateralised people were associated with commissural tracts and bilateral AF (n = 207; p < 0.01, FDR corrected). Abbreviations: AF, arcuate fasciculus; CC, corpus callosum. Download Figure 7-1, TIF file. [file jneuro-44-e0166242024-s002.tif]
